# Evaluation of focused antenatal care services quality at University of Gondar Comprehensive Specialized Hospital, Central Gondar zone, Northwest Ethiopia

**DOI:** 10.1371/journal.pone.0310038

**Published:** 2024-10-31

**Authors:** Tibebu Tadesse Fenta, Asmamaw Atinafu, Banchlay Addis

**Affiliations:** 1 University of Gondar Comprehensive Specialized Hospital, Gondar, Ethiopia; 2 Department of Health Systems and Policy, College of Medicine and Health Science, University of Gondar, Gondar, Ethiopia; CISM: Centro de Investigacao em Saude de Manhica / Instituicao Nacional de Saude (INS), MOZAMBIQUE

## Abstract

**Background:**

Antenatal care is the care that women receive during their pregnancy to ensure the health of the mother and her baby. The provision of information on birth preparedness in Ethiopia is still low, which implies identification of pre-existing health conditions that may affect the outcome of pregnancies was not provided. Even if the Focused Antenatal Care service was provided in our setup, maternal death was still high. In addition, as far as our search, the quality of antenatal care service in this facility has not yet been evaluated. As a result, the purpose of this study was to assess the quality of focused antenatal care services at the University of Gondar Comprehensive Specialized Hospital, Northwest Ethiopia.

**Methods:**

A mixed method case study evaluation design was employed at the University of Gondar Comprehensive Specialized Hospital from February 29 to March 29, 2020. Quantitative data was collected through exit interviews of 411 mothers who attended antenatal care follow-up and review of 422 clients’ cards 2 months prior to the study period. Similarly, 35 items of resources inventory, 10 direct observations, and 12 key informant interviews were conducted. The data were entered into Epi-Data version 4.6 and exported to SPSS version 25 and it was analysed with the Evaluation judgment matrix analysis method. Qualitative data were transcribed, translated and analysed by using thematic analysis. The evaluation focused on availability, compliance, and satisfaction dimensions with 25 indicators to judge the quality of antenatal care services.

**Results:**

The overall quality of focused antenatal care services was judged as good, based on pre-set judgment criteria **(76.7%),** to which availability of resources, healthcare provider’s compliance with the national guideline, and maternal satisfaction contributed, 73.3, 77.75, and 78.8%, respectively. Auditory and visual privacy were practiced which enhances patient confidentiality and personal dignity. The waiting area was perceived to be satisfactory by the majority (89%) of ANC clients, but there is no separate waiting area for ANC clients. Ten (10) client/provider interactions were observed, demonstrating that all clients were counseled about pregnancy danger signs.

**Conclusion and recommendation:**

The overall focused antenatal care services quality was relatively good, although the availability of resources was fair. Some essential drugs and medical equipment were out of stock. Therefore, the hospital had better fulfill crucial medicines and equipment for better service quality.

## Introduction

Globally, an estimated 295,000 maternal deaths occurred, yielding an overall maternal mortality ratio of 211 maternal deaths per 100,000 live births for the 185 countries and territories in 2017. Besides every day, approximately 810 women die from preventable causes related to pregnancy and childbirth [1]. Pregnant women who received quality antenatal care were 4.6 to 47.1% globally and 24.3% to 66.8% in Ethiopia [2–4]. Current Sustainable Development Goal (SDG) 3 target 3.1: aimed to reduce Maternal Mortality Ratio (MMR) below 70 per 100,000 live births at the end of 2030, which is the average global target [5]. Sub-Saharan Africa had the highest MMR in 2015, with an estimated 546 maternal deaths per 100,000 live births [6]. Ethiopia is one of the countries with the highest maternal mortality ratio, with 412 deaths per 100,000 live births and neonatal mortality of 29 deaths per 1,000 births according to the 2016 Ethiopian Demographic and Health Survey (EDHS) reports, of which most of the deaths were attributed to high-risk fertility behavior [7, 8]. The total fertility rate in 2016 was 2.5 and 4.8 per woman globally and in Ethiopia, respectively, showing that the world’s population is rapidly growing [9].

Improving maternal health is one of the global health agendas. Achieving safe motherhood requires several initiatives that have been taken in recent years to improve maternal health [1, 10]. Focused antenatal care was instituted in 2002 by the World Health Organization [10] to overcome the challenges posed by the traditional antenatal model of care such as classifying pregnant women into high-risk or low-risk groups based on pre-identified criteria, and the possibility of the low-risk group developing complications at delivery [11].

One of the global health priorities is to improve maternal health. Several recent measures to promote maternal health are needed in order to achieve safe motherhood [2].

Focused antenatal care (FANC) is an approach to ANC that emphasizes, individualized care, client- centered, fewer but comprehensive visits, disease detection not risk classification, and care by a skilled provider which focuses on the quality of Antenatal care rather than the frequency of visit [12].

Preventing, screening and treating infections during pregnancy prevents fetal loss, preterm delivery, low birth weight, maternal and infant morbidity and anti-tetanus immunization and prevention of mother-to-child-transmission of HIV (PMTCT) is known to protect infant health [13, 14].

The quality of antenatal care (ANC) is dependent on the qualifications of health care providers, the number and frequency of ANC visits, the content of services received, and the kinds of information given to women during their ANC visits [15].

According to Ethiopia’s health sector transformation for quality guidelines, all women who came for ANC follow-up should be routinely evaluated and given timely and appropriate care, all problems should be identified and senior health professionals consulted, blood pressure should be measured at each visit, correctly interpreted, and proper management should be given, and all necessary lab tests should be performed [16].

Good quality ANC improves maternal health and decreases the chances of suffering from anemia, pregnancy-induced hypertension, and preterm labor and promotes positive pregnancy outcomes, including a reduced risk of low birth weight (<2,500 grams) and preterm babies [17–20]. In addition to the number of visits, the components included in ANC greatly influence its effectiveness and might also affect women’s decisions regarding the time of initiation and continuity of care [21, 22]. Poor-quality ANC has the potential to reduce its use [23].

Globally, an estimated 295,000 maternal deaths occurred, yielding an overall maternal mortality ratio of 211 maternal deaths per 100,000 live births for the 185 countries and territories in 2017. Besides every day, approximately 810 women die from preventable causes related to pregnancy and childbirth [1]. Pregnant women who received quality antenatal care were 4.6 to 47.1% globally and 24.3% to 66.8% in Ethiopia [2, 3].

Previous studies attempting to assess FANC in the country took into account the frequency of ANC visits rather than the content of services and information provided to pregnant women during ANC visits [24]. In this respect, besides reporting the overall coverage, how many pregnant women receive standardized FANC services in Ethiopia is un clear [25, 26]. Although researchers often emphasize the importance of quality of maternal care in improving maternal and newborn health, the quality of FANCs remains too little studied [27, 28]. Besides, since up to the best knowledge of the evaluator the process of Quality of FANC has never been evaluated before at the University of Gondar Comprehensive Specialized Hospital.

Therefore, this study aimed to evaluate the quality of antenatal care services provision among pregnant women attending antenatal care at the University of Gondar Comprehensive Specialized hospital.

## Methods

### Evaluation setting and period

The Evaluation was conducted at the University of Gondar Comprehensive Specialized Hospital (UoGCSH) located in the Central Gondar zone of Amhara National Regional State, Northwest Ethiopia from February 29 to March 29, 2020. It is located 780 km away from Addis Ababa (capital city of Ethiopia). The hospital was established in 1954 as a public health college and training center and currently, it is one of the referrals and teaching hospitals serving more than 7 million people in the region through Gynaecology, Pediatrics, Internal Medicine, Surgery, Ophthalmology, Dermatology, Pharmacology, Psychiatry and Psychotherapy, MDR TB, Kaalzar, and Cancer Treatment center, Fistula, Pediatric Rehabilitation, and other social services. The hospital has 8 outpatient service units for Antenatal care services. The total number of clients who have got ANC service monthly was around 1646.

### Evaluation approach and dimensions

The formative evaluation approach using a single case study design was conducted to obtain detailed and explorative reports on FANC services quality. The quantitative and qualitative data were collected simultaneously, analyzed separately, and mixed during interpretations of the findings. This evaluation assesses the quality of FANC services using availability, compliance, and satisfaction dimensions based on the interests of stakeholders. The availability of resources was assessed using 08 item indicators to determine whether essential drugs assesses the quality of FANC services using availability, compliance, and satisfaction dimensions based on stakeholders’ interests and medical equipment required for the services were supplied or not in the hospital. The compliance of healthcare providers was also assessed using 07item indicators by measuring the adherence level of the healthcare providers to the national FANC guidelines during the diagnosis or classification and treatment of pregnant women. Moreover, maternal satisfaction was measured using 10-item indicators.

### Sample size and sampling procedures

Availability of necessary medical equipment, supplies and medicines, and human resources and infrastructure were observed by using a resource inventory checklist. The observation was conducted in the ANC service unit to assess the healthcare provider’s adherence to the national FANC guideline. To assess the compliance of one healthcare provider as per the national guideline observation data were collected until information saturation. The observation session represented the whole activity of the team and enabled us to assess and judge the strengths and weaknesses of healthcare providers as a whole or individuals. The compliance was also assessed using document review and the sample size was calculated using a single population proportion formula considering 48.3% proportion [2], 1.96 confidence level, 5% margin of error, and 10% incomplete data. The calculated sample size was 422and reviewed the maternal record from 29 February 2020 to 29 March 2020. A purposive sampling technique was used to select the key informants based on seniority (level of education) and position. The sample size for the satisfaction dimension was determined using a single population proportion formula considering 58% of patients satisfied by the service [12],95% confidence level, 0.05 margin of error, and 10% non response rate. Our final sample size for the satisfaction dimension was 411.

### Operational definition

**Availability:** it is one sub-dimension of access measurements used to assess the availability of program resources for the delivery of FANC services and measured by 08 item inventory assessment indicators. And finally, it was judged as Very good ≥85%, Good = 75–84%, Fair = 55–74%, and Poor (critical) = < = 54% [29]

**Compliance:** is the occurrence of diagnosis and treatment activities of FANC services based on National guideline recommendations. For this evaluation, it was measured by 07 item compliance indicators and judged as Very good ≥85%, Good = 75–84%, Fair = 55–74%, and Poor (critical) < = 54% [29].

**Client satisfaction:** this can be defined as the extent of an individual’s experience compared with his or her expectations. For this evaluation, it was measured by 10-item satisfaction indicators and judged as Very good ≥85%, Good = 75–84%, Fair = 55–74%, and Poor (critical) = < = 54% [29].

**Focused Antenatal Care Service Quality:** For this evaluation study defined as a care that is provided to pregnant women during pregnancy emphasizing on availability of resources for service delivery, health care provider’s adherence to national standards, and improvement of women’s satisfaction. Finally, it was judged based on the performance of the final result of the evaluation. With some evidence and agreement with stakeholders, five ordinal scales with their values were selected to judge the Evaluation of FANC service quality, very good ≥85%, Good = 75–84%, Fair = 55–74%, and Poor (critical) = < = 54% [29].

### Data collection tools and procedures

The semi-structured questionnaire, interview guide, resource inventory, observation, and data extraction checklist were prepared by reviewing of literatures [30, 31]. Indicators were also developed from the national FANC guideline, BEmONC guideline, and other related evaluations [2, 31, 32]. Semi-structured interviewer administered questionnaire containing the background characteristics, reproductive history, accessibility, and acceptability of the service. Ten (10) direct observations with an observation checklist were used to assess the healthcare provider’s -client interactions and providers’ adherence to FANC national standards, including the interpersonal interaction, ways of provider’s history taking, information transfer, and other components of FANC as per the national standards offered to FANC clients. Thirty-five items resource inventory checklist was used to assess the existence of the required resources used for FANC service. This tool contains infrastructures, human resources, laboratory diagnostic tests, essential drugs, and medical equipment/supplies for FANC services. The interview guide was prepared for key informants, such as the school of Gynaecology Head, Clinical Coordinator, Midwifery Coordinator, MCH Head, health care providers, and focused on assessing availability; compliance and satisfaction. A data extraction checklist tool was prepared to review the specific procedure of diagnostic and medical supplies from the stock balance card and patients’ folder. The questionnaire and interview guidelines were translated into the local language (Amharic) and then translated back to English language to ensure consistency.

Six health professionals, (3 diploma and 2 BSc midwives) and one MSc midwife as a supervisor who have experience of data collection before were recruited. For the data quality, quantitative data were collected by principal investigator and MSc Midwife who have experience on collecting qualitative data before. Data collectors including the supervisor were trained together for two days.

The drug store, FANC room, laboratory room, and the whole physical working environment via the resource inventory checklist were assessed to evaluate the availability of resources needed for FANC service two months (January to February 2020) prior to data collection in the respective units at University of Gondar comprehensive specialized hospital. The availability of resources was also checked by interviewing the team leader of MCH, laboratory coordinator and pharmacy coordinator of the hospital. The exit interview was carried out for mothers who came for FANC follow-up to assess satisfaction. For compliance, direct observation was conducted at the FANC service room after the principal evaluator got informed consent from the coordinators. For qualitative data, 12 key informant interviews (KIIs) were conducted by the principal evaluator after getting informed consent from interviewees. Key informants were interviewed using the interview guide and probing was done following their response to receive more information. A tape recorder and taking field notes were required during the interviewing process. The interviews were commonly taking place at their offices and it takes a time range of 35 to 60 minutes with an average of 42 minutes duration.

### Data quality control

Two days training were given for data collectors about the basic techniques of data collection. A pre-test was also conducted on 21(5%) participants at Felegehiwot Specialized Hospital, Bahir Dar, Ethiopia, and necessary modifications were made. Observation, data extraction, and resource inventory checklists were pretested and amendments were made. The questionnaire was checked for its completeness on a daily basis by the principal evaluator and supervisors during data collection. The principal evaluators transcribe the voices of the respondents into text to analyze and check the consistency of the information with the initials. Qualitative data was gathered, transcribed into the text format of Amharic, and translated back into the English language by another translator to see the internal consistency. In addition, during observation the healthcare providers did not know who observed them and it was done by investigators after the principal evaluator had got permission from the hospital manager.

### Data management and analysis

The quantitative data were cleaned and checked for completeness, consistency and coded by the trained supervisor and principal evaluator. Data were entered into Epi data version 4.6 Software and exported to SPSS version 25 for analysis. The qualitative data was analyzed using Open code software 4.02 under the thematized area to supplement quantitative findings.

Availability, compliance and satisfaction dimensions of quality of FANC program were evaluated and judged as Very good, Good, Fair, and Poor with evaluation judgment matrix analysis.

### Judgment matrix

The weight of each dimension of the FANC program was determined by the agreement of the stakeholders. Value was given for each dimension proportionally according to the level of importance considered by the stakeholders. The score of each dimension was aggregated to measure the quality of FANC program based on the predetermined judgment criteria. Indicators’ weight is the weight given by stakeholders before the evaluation for each selected indicator, and indicator scores were calculated using the formula (Indicator score = ((observed X weight)/Expected) [2, 32–34]. The weighted values of availability, compliance, and satisfaction were 32, 28, and 40%, respectively. The judgment parameter for each dimension and the overall program quality were also categorized as Poor, Fair, Good, and Very good with the corresponding judgment values of; < 65%, 65–74.9%, 75–90%, and > 90% respectively.

### Ethical approval and consent to participate

Before the commencement of the study, ethical clearance was obtained from the institutional review board of the University of Gondar College of Medicine and Health sciences, Institute of Public Health (Ref. No. IPH/837/6/2012). Then permission letter was secured from the University of Gondar Comprehensive and Specialized Hospital before starting data collection. For age ≥ 18 years informed written consent was obtained from study participants after a brief explanation of the purpose, benefit, and risk of the evaluation in a language that a potential participant could understand prior to participation. While those who were <18 years of age gave via their families/attendants prior to the data collection process. After data collectors provided complete information to illiterate participants, they signed with their fingers after they understood and agreed to participate. For illiterate participants, a non-research staff member (Midwives) or a patient’s relative served as an impartial witness throughout the informed consent process.

To ensure confidentiality, names were not used instead coded numbers were assigned to depict the results and the questionnaires were kept locked.We assure that the methods are performed with the declaration of Helensiki.

## Results

### Socio-demographic characteristics of the study population

A total of 411 ANC clients were interviewed. The mean age for respondents was 26.2 (SD ±3) years with a range of 17 to 37 years. About 93.7% of mothers were married, 83.9% were Orthodox Christian, 64.2% were collage and above and 58.4% were housewives (Table 1).

**Table 1:** Socio-demographic characteristics of mothers at UOGCSH, Central Gondar Ethiopia, May 2020. (n = 411).

Variables	Frequency (n)	Percent (%)
Age in years		
<20	10	2.4
20–24	98	23.8
25–30	279	67.9
>30	24	5.9
Marital status		
Married	382	92.9
Single	10	2.4
Widowed	6	1.5
Divorced	13	3.2
Educational status		
Unable to read and write	29	7.1
Able to read and write without formal education	19	4.6
Grade 1–8	42	10.2
Grade 9–12	57	13.9
Collage and above	264	64.2
Occupational status		
Government employee	70	17.0
Merchant	59	14.4
Daily labor	15	3.6
Farmer	22	5.4
Housewife	240	58.4
Private employee	5	1.2
Religion		
Orthodox	345	83.9
Muslim	59	14.4
Protestant	7	1.7
Residence		
Urban	372	90.5
Rural	39	9.5
Gravidity		
Primigravida	89	21.7
Multigravida	318	77.4
Grand multipara	4	0.9
Parity		
Have no child	97	23.6
One child	221	53.8
Two to four children	93	22.6
Frequency of visits		
First-visit	63	15.3
Follow-up visit	348	84.7

### Availability of resources (input)

An observation made on the level of auditory and visual privacy of the ANC/PMTCT room found that auditory and visual privacy were practiced which enhances patient confidentiality and personal dignity. The waiting area was perceived to be satisfactory by the majority (89%) of ANC clients, but there is no separate waiting area for ANC clients so, they share waiting areas with other clients. There were five functional FANC rooms and there was running water in each room with soap.

Based on the resource inventory checklist and key informant responses, there were 15 obstetricians,50 residents,4 General Practitioners (GPs), additional intern students and also 16 midwives working in the ANC clinic, but no one was trained on FANC/PMTCT for the last three months.

‘‘Reasons mentioned by major key informant respondents were a shortage of budget and lack of support from NGOs the fact that the Hospital supports part of the expenses, which is not enough to cover the high client demand. Drug shortages were a national issue. We made numerous attempts to purchase. Nevertheless, there is no money now because of the COVID-19 pandemic, and we’ve canceled two purchasing processes. For instance, cotrimoxazole is a drug that is often discontinued and is pricey when it is purchased. Furthermore, we are unable to complete any task at once because the facility is the only tertiary hospital in the central Gondar zone.” [28 years old female HCP working at UOGCSH].

During site observation, FANC guideline (BEmONC) and Management Protocol on Selected Obstetrics Topics were observed in the ANC clinic and EHSTG and HSTQ guidelines in the quality improvement office. Findings from the inventory checklist showed that ANC equipment like fetoscopy, weighing scale; BP apparatus and ultrasound were observed at the ANC clinic and all of this equipment was functional. FANC essential medicines like iron folate and Tetanus Toxoid were 96% available and there was no stock out reported during the last three months. But for some medicines like Anti-D for Rh-ve women and cotrimoxazole and plumpy nut for HIV -exposed infants and HIV +ve women was a stockout reported during the last three months and it was not available during the study period (Table 2).

**Table 2:** Summary of performance of availability indicators for FANC service at UOGCSH, Central Gondar Ethiopia, May 2020.

No	Availability	E*	O*	W*	IS*	A*	J*
1	Availability of ANC rooms with fully functional comprehensive FANC services	5	5	4.2	4	83	Good
2	Availability of trained health professionals on FANC services/PMTCT for the last three months	16	0	4.2	0	0	Poor
3	Availability of key essential drugs without stock out for the last three months (Iron folate, TT, HAART, PT, antihypertensive, anticonvulsant, antibiotics, Mebendazole, cotrimoxazole….)	11	8	4	2.91	80	Good
4	Availability of equipment/supplies without stock out for the last three months (BP apparatus, weight scale, Fetoscope, statoscope, and others)	21	17	4	3.24	81	Good
5	Availability of IP materials (7 items)	7	3	3.2	1.37	43	Poor
6	Availability of ANC guidelines at FANC rooms (FMOH or WHO…)	5	5	4	4	83	Good
7	Availability of standard recording and reporting formats	5	5	4	4	100	V.good
8	The proportion of ANC rooms with functional pipe water	5	5	4	4	100	V.good
	Overall availability			32	23.52	73.5	Fair

*E: Expected, O: Observed; W: weight, S = Score ((observed X weight)/Expected), A: Achievement in percentage = ((S/W) * 100), J: Judgments.

‘‘Because some materials and drugs related to MCH services which are usually covered by the hospital’ so that sometimes there is a shortage of budget and also there is a shortage of drugs at Ethiopian Pharmaceuticals Supply Agency (EPSA) level”. [34 years old male HCP working in UOGCSH].

“Possible reasons were a shortage of budget to cover the cost and also most of the time reagents and drugs run out before the expected time because the number of reagents and drugs requested mismatch with the number of reagents needed in reality and sometimes some drugs were abused like Anti-D” [34 years old male HCP working in UOGCSH].

The FANC clinic had an almost complete set of routine laboratory reagents for ANC client tests. However, reagents for HIV tests were not available during the study period.

‘‘The possible reason for the absence of HIV kits in the FANC clinic was due to the expiration date. Since HIV test kits were obtained from NGOs by donation, it was difficult to replace them immediately.” [36 years old male HCP working in UOGCSH]

The implementation level of the hospital concerning the availability of resources according to facility inventory and an expert interview was judged to be fair (73.5%, (Table 2).

### Compliance with FMOH/WHO standards

To assess ANC service provision compliance with WHO standards, 422 ANC client cards were reviewed, and 10 client-provider interactions were observed. Compliance with the standard was measured using a scale derived from the key components of WHO focused Antenatal Care; comprehensive history taking, detection of existing diseases and management of complications, prevention of disease and promotion of health, birth preparedness, and prevention of complications.

This evaluation finding revealed that the process of the FANC clinic about compliance with the WHO standard was judged to be good (77.75%), as shown in (Table 3).

**Table 3:** Summary of performance indicators compliance to national FANC service guidelines at UOGCSH, Central Gondar Ethiopia, May 2020.

No	Compliance	E*	O*	W*	IS*	A*	J*
1	The proportion of FANC clients who gate service with appropriate time	422	157	3.5	1.3	37	Poor(critical)
2	The proportion of FANC clients who measured and recorded blood pressure	422	403	4	3.82	96	Very good
3	The proportion of FANC clients who received VDRL testing and counseling	422	403	4	3.82	96	Very good
4	The proportion of FANC clients who received urinalysis testing	422	403	4	3.82	96	Very good
5	The proportion of FANC clients who received hemoglobin/hematocrit testing	422	403	4	3.82	96	Very good
6	The proportion of FANC clients who received HIV testing and counseling	422	132	4.5	1.41	31	Poor(critical)
7	The proportion of FANC clients who received Iron folate and TT vaccine	422	399	4	3.78	95	Very good
	Overall compliance			28	21.77	77.75	Good

*E: Expected, O: Observed, W: weight, S = Score ((observed X weight)/Expected), A: Achievement in percentage = ((S/W) * 100), J: Judgement.

‘‘Out of the standards, guidelines, and audit tools developed by FMOH, one of the chapters focuses on maternal and child health. For example, the Ethiopian Hospitals Services Transformation Guideline (EHSTG) has around 20 chapters, from this chapter 8 is only focused on maternal and child health. So, we did our work in the MCH clinic and other maternal units based on this guideline. To check whether or not the HCPs were working on this guideline, each quarter our QI teams developed QI audits. As an example, a client survey was conducted at the FANC clinic. During that presentation, clients stayed more than 24hours due to laboratory results delay, so we immediately developed Quality Improvement Project (QIP) and availed a side laboratory at the FANC clinic and decreased clients’ stay from 24hrs to 6hrs.” [38 years old male HCP working in UOGCSH].

#### Comprehensive history taking

For the majority of 298(71%) ANC clients, a comprehensive history were recorded on their cards. On the other hand, 10 first visit client-provider interactions were observed and comprehensive history was taken for all clients.

#### Physical examination

Routine physical examinations appropriate for the first ANC visit were recorded on the client’s card, weight 410(97%), BP 410(97%), uterine height 406 (96%), and pallor 410 (97%) of participants. From ten (10) first-visit client-provider interactions 09 of them got routine physical examinations appropriate for their visit but uterine height and conjunctiva (sclera examination) were not done for one client.

#### Routine laboratory investigation

Routine laboratory investigations appropriate for the first ANC visit, VDRL test for 403(96%), hemoglobin test for 402(95%, Blood group, and RH test for 403(96%), HIV test for 132(31%), and stool examination for 391(93%) of clients were performed. Ten (10) first-visit client-provider interactions were observed and all of them were tested for hemoglobin, VDRL, blood group and Rh and stool examination, but none of them were tested for HIV test during the study period. Findings from the inventory tool showed that there was stock out of reagents for HIV tests for the last three months. The hospital provided all types of laboratory investigations required for antenatal care services but from reviewed client cards it was found that HIV tests were not performed, out of 422 cards reviewed only 132 (31%) clients got the test.

‘‘At the movement, our laboratory services have been relatively good, we have a side laboratory at the ANC clinic, but due to a lack of reagents, sometimes necessary tests have been done out of the hospital.” [42 years old male HCP working in UOGCSH].

“Currently we haven’t an HIV test kit due to expiration date. Due to this, we can’t provide HIV testing. Besides, even if we have a side laboratory with some essential laboratory reagents at the FANC clinic, it is not standardized. It is only to reduce the client’s load and discomfort, and minimize waiting time. Otherwise, the room is very narrow and everything is done in this room. Due to that, their health care providers were exposed to HIV and they take prophylaxis before three months.” [26 years old male HCP working in UOGCSH*]*.

#### Prevention of diseases and promotion of health

From 422 reviewed cards 395(94%) of clients received a prescription for Iron supplementation, 403(96%) of clients were referred for TT vaccination, and 273 (65%) of clients were counseled for ITN utilization. Results from client-provider interaction observations revealed that in ten (10) session’s clients were referred for tetanus toxoid vaccination and received a prescription for Iron supplementation. However, only 4 out of 10 clients were counseled for ITN utilization. Findings from resource inventory and expert interviews showed that there was stock out of Anti-D, Cotrimoxazole, and plumpy nut during the last three months even at zonal and regional levels.

### Client education and counseling on nutrition and danger signs of pregnancy

WHO focused antenatal care guidelines recommend eight items of advice for pregnant women during their first visit. From ten (10) first visit client-provider interactions observed none of the clients got counseling on such items as use of ITN, nutrition, hygiene, and FP (birth spacing). However, all clients received counseling on pregnancy danger signs.

“Since health centers in the town were not exercise MCH services as expected, we usually provide all services without referral in this clinic including PMTCT services, and this hospital not only serves Gondar town but other clients of the neighboring district with high client follow and which is range from 150 to 200 clients per day”. Even if client flow were very high, we try our best to provide services for the clients, but it is difficult to provide counseling (health education) to all clients, especially in a group and quality services, this has a strong effect on client satisfaction and quality of care because we can’t give enough time for the mother due to work overload.” [27 years old female HCP working in UOGCSH].

#### Birth preparedness and complication readiness (danger sign of pregnancy)

Only 135 (32%) and 410(97%) of the clients received counseling on birth preparedness and pregnancy danger signs respectively. From ten (10) client–provider interactions it was observed that all clients were counseled about pregnancy danger signs, but none of them were counseled for birth preparedness.

‘‘I think there is a problem with the level of adequacy of explaining the dangerous signs of pregnancy to mothers. For example, here in Kebele 16 (Chechela) near to our hospital, I remember one case, where her membrane was ruptured and she stayed at home for 3 days and her baby died since the cord was expelled. This indicates there was a major gap in danger sign counseling. Besides, there is also a gap in family planning counseling. A woman who came for ANC visit has the right to get FP counseling and to decide her choice to take immediately after birth. But there is a big gap in women’s advice about family planning in the case of family health and economic aspects. I think this is a missed opportunity. Otherwise, I believe that other works are going well.” [42 years old male HCP working in UOGCSH].

By aggregating indicator scores, the process of the FANC clinic about compliance to WHO standard was judged to be good (77.75%), as shown in Table 3.

#### Clients’ satisfaction towards the quality of FANC

A total of 411 ANC clients were interviewed soon after their completion of the day’s service. 74 (27.9%) of clients, 116 (43.8%), 52 (19.6%), 21 (7.9%), and 2 (0.8%) of clients attended first, second, third, fourth, and above fourth visit respectively

The result indicated that 224 (84.5%) of the respondents were satisfied with the adequacy of working hours and 229 (86.4%) were satisfied with the time spent with the provider. All (100%) of them perceived that the appointing system for follow-up is convenient while 143 (54%) were satisfied with the convenience of the waiting area. The clients ascertained that the average waiting time spent for routine antenatal follow-up to see a service provider was 29 minutes but 133 (50.2%) of the respondents waited for less than 29 minutes. Majority, 185 (69.8%) said that the consultation time with a provider was for less than 15 minutes and only 80 (30.2%) said that it was greater than 15 minutes (Table 4).

**Table 4:** Summary of performance of satisfaction indicators for FANC service at UOGCSH, Central Gondar Ethiopia, 2020.

No	Satisfaction	E*	O*	W*	S*	A*	J*
1	The proportion of pregnant mothers who have satisfied within being able to get good FANC when they needit	411	377	4	3.67	90.5	Very good
2	The proportion of pregnant mothers satisfied with their information about where to get the FANC	411	393	4	3.82	97	Very good
3	The proportion of pregnant mothers satisfied with the availability of side laboratory service	411	402	4	3.91	98	Very good
4	The proportion of pregnant mothers satisfied with the availability of essential drugs	411	395	4	3.84	96	Very good
5	The proportion of pregnant mothers satisfied with the availability of equipment	411	171	4	1.66	42	Poor(critical)
6	The proportion of pregnant mothers satisfied with the appearance of health professionals	411	403	4	3.92	98	Very good
7	The proportion of pregnant mothers satisfied with pregnancy danger signs advice?	411	403	4	3.92	98	Very good
8	The proportion of pregnant mothers satisfied with birth preparedness and complication readiness counseling?	411	134	4	1.30	33	Poor(critical)
9	The proportion of pregnant mothers satisfied with the free service of the entire program	411	406	4	3.95	99	Very good
10	The proportion of pregnant mother’s satisfied with the overall time spent to get the service	411	157	4	1.53	38	Poor(critical)
	Overall satisfaction			40	31.52	78.8	Good

*E: Expected, O: Observed, W: weight, S: Score ((observed X weight)/Expected), A: Achievement in percentage ((S/W) * 100), J: Judgment.

### Overall quality

The overall quality of FANC service at the University of Gondar Comprehensive and Specialized Hospital is 76.7% and satisfaction accounts for 32% of the total (Table 5)

**Table 5:** Summary of dimensions for overall FANC service quality at UOGCSH, 2020.

No	Dimension	Expected	Observed	Indicators	Weight	Result	Judgment
1	Availability	100%	73.5%	08	32%	23.52	Fair
2	Compliance	100%	77.75%	07	28%	21.77	Good
3	Satisfaction	100%	78.8%	10	40%	32	Good
Overall quality of the FANC service				25	100%	76.7%	Good

## Discussion

This study aimed to evaluate the quality of focused antenatal care services at the University of Gondar Specialized Comprehensive Hospital, Northwest Ethiopia. The overall quality of focused antenatal care services was judged as good (76.7%), to which availability of resources, healthcare provider’s compliance with the national guideline, and maternal satisfaction contributed, 73.5, 77.75, and 78.8%, respectively.

This result is higher than the study conducted on the Quality of Prenatal Care and Associated Factors among Pregnant Women at Public Health Facilities of Wogera District, Northwest Ethiopia, 32.7% [35]. The differences might be due to measurement variation and setup, specialized hospitals might have special care than health centers, primary or general hospitals.

It is also slightly higher than the study conducted at Higher 2 Health Center in Jimma Zone, Oromia Region South West Ethiopia, 69.5% [2]. The differences might be related to the difference in setup, hospital setup may have higher quality than health center, and some indicators difference.

The results of this evaluation revealed that ANC equipment like fetoscopy, weight scale; BP apparatus, and WHO focused ANC standard guidelines Iron folate and tetanus toxoid were available at ANC clinic. This was found to be in line with the recommendation of focused ANC standards and study conducted on the implementation of Focused Antenatal Care Service in Agaro Health Center, Jimma Zone, Oromia Region South West Ethiopia [32, 36].

However focused ANC service is implemented in the hospital with a shortage of basic resources like height scale, fundal height measurements, IP supply, IEC materials. This result is contradicted with the recommendation of focused ANC standards. The reason might be due to a lack of budget in this set up.

This evaluation also showed a shortage of trained staff and some essential drugs like anti-D and cotrimoxazole, and it is similar to a study conducted on Focused Antenatal Care Service in Agaro Health Center, Jimma Zone, Oromia Region South West Ethiopia [32]. The reason might be due to a lack of budget and drugs as a national issue because of the COVID-19 pandemic and lack of appropriate planning at the beginning, drugs requested mismatch with the number of reagents needed due to high client flow. The other reason might be that the drug suppliers in the country are very limited, their remote location from the institutions and high transportation costs. In addition, because the medical facilities in the country are limited the number of users is greater than the catchment population which leads to the drugs running out quickly.

In general, the overall availability of resources was judged as fair based on pre-set judgment criteria. This result is almost similar to the study conducted in Jimma [2]. The possible similarity might be both studies used almost similar indicators for measurement.

In this evaluation provider, the compliance level to the WHO focused ANC standard was found to be good based on the pre-set evaluation judgment criteria. This result is slightly higher than the study conducted at Higher 2 Health Center in Jimma Zone, Oromia Region South West Ethiopia. The differences might be related to the difference in the setup, the hospital setup might usestandard guidelines than a health center [2]. This result is also higher than the study conducted on the implementation of Focused Antenatal Care Service in Agaro Health Center, Jimma Zone, Oromia Region South West Ethiopia43% [2]. The differences might be due to setup differences, some indicator differences, and the year of study.

Comprehensive history taking, detection of existing disease and complications, prevention of disease and promotion of health, and birth preparedness and complication readiness are WHO standards of focused antenatal care services [36].This evaluation result revealed that a complete history was recorded on ANC clients’ cards for the majority of the client’s, 71%. This result is lower than WHO standards and slightly higher than a study conducted on the Quality of Antenatal Care Service Provision and Associated Factors at Governmental Health Facilities of Harar Town, Eastern Ethiopia, 68.1% [3]. The difference might be due to setup differences, study design, and sample size variation.

Besides this result is lower than the study conducted at Higher 2 Health Center in Jimma Zone, Oromia Region South West Ethiopia [2]. The reason might be measurement difference, in a previous study there were four measurements used, whereas in this evaluation we used seven measurements to measure complete history taking.

All pregnant women should be detected for existing diseases and complications (physical examination and laboratory investigation) as recommended in WHO standards [36]. In this evaluation, only four routine physical examinations appropriate for the first visit were recorded on the ANC client’s card, weight 99.8%, BP 99.8%, uterine height 98.8%, and pallor 99.8%. From ten (10) first visit client-provider interactions 09 of them got routine physical examinations appropriate for their visit but, uterine height was not done for one client. This result is lower than WHO standards. The lower performance of WHO standards might be due to work overload.

Routine laboratory investigation for the prevention of common diseases in pregnancy and promotion of health during their first visit should be given for all pregnant women [36]. In this evaluation, routine laboratory investigations appropriate for the first visit (urinalysis, VDRL, Hemoglobin, HIV test, blood group, and Rh), and additional stool examination were done. HIV test was done only for 31% of pregnant mothers which is much lower than a similar study conducted on the Quality of Antenatal Care Service Provision and Associated Factors at Governmental Health Facilities of Harar Town, Eastern Ethiopia, 2017 [3]. The differences might be due to the lack of an HIV test kit because of the expiration date.

Ten (10) first- visit client-provider interactions were observed and all of them were tested for hemoglobin, VDRL, blood group, and Rh and stool examination, but none of them were tested for HIV test during the study period. This result contradicts WHO standard. The low performance might be due to the lack of HIV test kits because of the expiration date.

Prevention of anemia and tetanus in pregnancy: From 422 reviewed cards 94%of clients received a prescription for Iron supplementation, 98.1%of clients were referred for TT vaccination and66.4%of clients were counseled for ITNs utilization. This result is higherthan the study conducted at Higher 2 Health Center in Jimma Zone, Oromia Region South West Ethiopia [2]. The reason might be setup differences and sample size variation.

Results from client-provider interaction observations revealed that in 10 session’s clients were referred for tetanus toxoid vaccination and received a prescription for Iron supplementation. This result showed higher performance than two previous studies study conducted in Jimma [2, 32]. The reason might be setup differences; specialized hospitals give attention to special care.

However, only 4 out of 10 clients were counseled for ITN utilization. This is contradicting WHO standards. The reason might be a lack of counseling time due to work overload.

The focused ANC guideline recommends a counseling room with doors and windows to ensure auditory and visual privacy for ANC/PMTCT clients [36]. An observation found that there was a room for ANC/PMTCT counseling with windows and doors. However auditory and visual privacy was not practiced as the counseling session was observed to proceed at one point while another provider was examining another client within the same room. This result is in line with the study conducted on Process Evaluation of HIV Prevention of Mother to Child Transmission Program (PMTCT) in Agaro Health Centre, Jimma Zone, Oromia Region [29]. The reason might be due to work overload.

### Strength and limitation

Our work used three dimensions to evaluate the quality of FANC service which makes it more valid than measuring the quality by a single dimension. Besides, using both qualitative and quantitative methods (triangulation) also helped us get accurate and detailed results.

The possible limitations of the study are

⇒ Since it was a single case study design there were little basis for generalization of results to the wider population and Researchers’ own subjective feeling may influence the case study (researcher bias). And also, during the data collection time hawthorn effect (observation bias), sampling unit selection bias in case of observation might be overestimate or underestimate the evaluation findings.

## Conclusions

Generally, focused antenatal care service quality at the UOGCSH was relatively good, although the availability of resources was fair. Health care providers’ adherence with national FANC guidelines and client’s satisfaction were judged as good according to the pre-set judgment parameter. Thus, it is recommended that the hospital had better to give attention on availability of reagents or materials, and conduct regular resource inventory to prevent stakeouts.

In addition, the Hospital had better to balance antenatal care service providers to clients based on National standards to minimize workload. The Federal and regional Ministry of Health Bureau had better carry out regular support in order to maintain and improve the quality of FANC

## Supporting information

S1 Fig Logic model. (PDF)

S1 Table English version data collection tool. (PDF)

S1 File SPSS, STATA, and EXCEL Socio-demographic data. (RAR)

S2 File SPSS,STATA, and EXCEL availability dimension data. (RAR)

S3 File SPSS, STATA, and EXCEL compliance dimension data. (RAR)

S4 File SPSS, STATA, and EXCEL satisfaction dimension data. (RAR)

S5 File Ethical letter English version. (PDF)

## Abbreviations

AMDD: Averting Maternal Death and Disability
ANC: Antenatal Care
ARR: Annual Rate of Reduction
BEmONC: Basic Emergency Obstetric and Newborn Care
DHS: Demographic and Health Survey
FANC: Focused Antenatal Care
HCPs: Health Care Providers
HIV: Human Immunodeficiency Virus
LDCs: Least Developed Countries
MMR: Maternal Mortality Ratio
PMTCT: Prevention of Mother-To-Child-Transmission
SDG: Sustainable Development Goal
UOGCSH: University of Gondar Comprehensive Specialized Hospital
WHO: World Health Organization
