# Supplementary material for: Evaluation of focused antenatal care services quality at University of Gondar Comprehensive Specialized Hospital, Central Gondar zone, Northwest Ethiopia
Source: PLoS One. 2024 Oct 31;19(10):e0310038. doi: 10.1371/journal.pone.0310038 (PMC11527168; doi:10.1371/journal.pone.0310038)
Supplement: S1 Fig — (PDF) [file pone.0310038.s001.pdf]

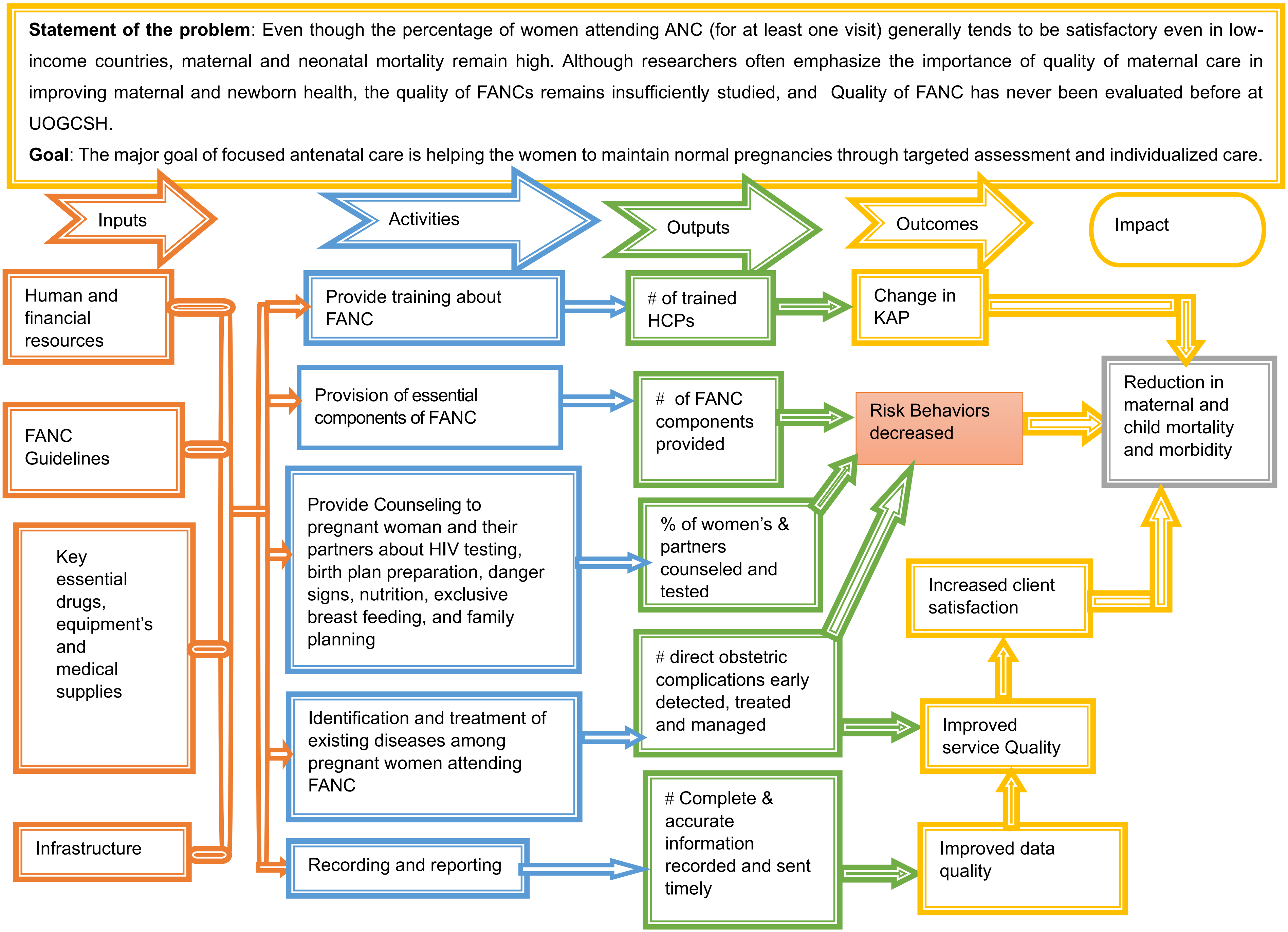

Figure 1: Logic Model of FANC adapted from MCH logic model at University of Gondar Comprehensive Specialized Hospital, Central Gondar Zone, 2020.
