# Supplementary material for: Evaluation of focused antenatal care services quality at University of Gondar Comprehensive Specialized Hospital, Central Gondar zone, Northwest Ethiopia
Source: PLoS One. 2024 Oct 31;19(10):e0310038. doi: 10.1371/journal.pone.0310038 (PMC11527168; doi:10.1371/journal.pone.0310038)
Supplement: S1 Table — (PDF) [file pone.0310038.s002.pdf]

Measurement tools (English version Questionnaires)

Quantitative data collection tools

Part1: Respondent's (pregnant mothers) Background

**Part 1: General information**			
**Q #**	**Questions**	**Response**	**Skip**
101.	Code	**--------------**	
102.	Age of the mother	**--------------**	
103.	Marital status	1. Married<br>2. Single<br>3. Widowed<br>4. Divorced<br>**77.** Other (specify)-------------	
104.	Educational status?	1. Unable to read and write<br>2. Able to read and write<br>3. Grade 1-4<br>4. Grade 5-8<br>5. Grade 9-12<br>6. Collage and above	
105.	Occupational status?	1. Gov't employee<br>2. Merchant<br>3. Daily labor<br>4. Farmer<br>5. Housewife<br>**77.** Others(specify) ____	
106.	What is your religion?	1. Orthodox<br>2. Muslim<br>3. Protestant<br>**77.** Other (specify)--------	
107.	What is your residence?	**1.** Urban<br>**2.** Rural	
108.	Type of visit:	--------------	
109.	Gravidity	-------------------	
110.	Number of delivery (both live and stillbirth after 28 weeks)?	------------------	
111.	LMP	*D/M/Y*-----------------	
112.	Gestational age at the initial visit	------------------*(weeks/)*	

Measurement tools (English version Questionnaires)

No	Part 2: Availability indicators measurement questions	Answers	
		Yes	No
201.	Does the hospital have fully functional comprehensive FANC services rooms?		
202.	Does the hospital have trained health professionals on FANC services in the last three months?		
203.	Does the hospital have essential drugs without stock out for the last three months (Iron folate, TT, HAART, PT, antihypertensive, anticonvulsant, antibiotics, Mebendazole, cotrimoxazole, deworming, STI drugs)?		
204.	Does the hospital have equipment/supplies without stock out for the last three months (U/S, BP apparatus, weight scale, Fetoscope, statoscope, and MUAC measurement)?		
205.	Does the hospital have IP materials (7 items)?		
206.	Does the hospital have ANC guideline at FANC rooms (FMOH or WHO…)?		
207.	Does the hospital have standard recording and reporting formats?		
208.	Does the hospital have ANC rooms with functional pipe water?		

Additional availability measurement questions

Inventory tool (Available =1, Not available =0)				Remark
		For the last 3 months	During data collection	
209.	Health professional trained(update) on FANC/PMTCT			
210.	Iron folate			
211.	TTV vaccine			
212.	Anti-D for Rh-ve women			
213.	ART prophylaxis/HAART			

214.	Cotrimoxazole for HIV exposed infants			
215.	PT for HIV +ve women			
216.	Opportunistic infection drugs (mebendazole...)			
217.	Antihypertensive drugs (hydralazine, pethidine…)			
218.	Anticonvulsants (MgSo4, Diazepam)			
219.	Antibiotics (Amoxicillin,)			
220.	STI drugs (Byzantine pencillike……)			
221.	Deworming			
222.	Functional fetoscope			
223.	Functional statoscope			
224.	Functional BP cuff			
225.	Functional Ultrasound			
226.	Tape meter (fundal height measurement)			
227.	Functional weight scale			
228.	Functional height scale			
229.	Examination table			
230.	Test kit for pregnancy			
231.	Test kit for HIV			
232.	Disposable syringe			
233.	Sharp box			
234.	Personal protective equipment (Cape, eye, google, face mask, glove, apron, boots)			
235.	Running water			
236.	Hand washing items(soap/detergent)			
237.	Client card			

238.	FANC Guideline/manual at each unit			
239.	IEC material			
240.	ANC registration book			
241.	ANC tally sheet			
242.	ANC recording & reporting format			
243.	ANC referral format			

**No**	**Part 3: Process/Compliance indicators measurement questions**	**Answers**	
		**Yes**	**No**
301.	Did the pregnant women gate service with appropriate time?		
302.	Does HCPs measure and recorded blood pressure?		
303.	Does clients received VDRL testing and counseling?		
304.	Does clients receive urinalysis testing?		
305.	Do clients receive hemoglobin/hematocrit testing?		
306.	Does clients receive HIV testing and counseling?		
307.	Does clients receive Iron folate and TT vaccine?		

Additional process measurement Questions

Checklist to observe HCPs Provision of essential components of FANC

History taking, Physical examination, laboratory examinations, Provision of therapeutics, Information provided and advice on danger signs for HCPs practice and client charts as needed will be observed.

**Provision of essential components of FANC**	Yes=1	No=0	Skip
**308. History taking**			
308.1. Personal history			
308.2. Social History			
308.3. Family history			
308.4. Medical history			

308.5. Surgical history			
308.6. Obstetric history			
308.7. History for current pregnancy.			
**309. Physical examination**	Yes=1	No=0	Skip
309.1. Weight			
309.2. Height			
309.3. BP			
309.4. Eye conjunctiva and sclera examination			
309.5. Neck examination			
309.6. Breast examination			
309.7. Abdominal examination			
309.8. Fetal heart rate recording			
309.9. Vaginal examination, if GA≥36 weeks			
309.10. Leg examination for edema and varicose vein examination.			
**310. Laboratory examination**	Yes=1	No=0	Skip
310.1. Hemoglobin/Hematocrit			
310.2. VDRL			
310.3. Blood group			
310.4. RH factor			
310.5. Urine test			
310.6. HIV test			
310.7. 7. HBsAG/HCAG			
310.8. Stool examination.			
**311. Provision of therapeutics**	Yes=1	No=0	Skip
311.1. Prophylactic iron/folate supplementation			
311.2. Tetanus toxoid administration			
311.3. ITN as needed			
312. **Information provision on birth preparedness and complication readiness**	Yes=1	No=0	skip

Measurement tools (English version Questionnaires)

312.1. Birth preparedness			
312.2. Place of birth			
312.3. Saving Funds			
312.4. Potential blood donors in case of emergency			
312.5. Birth spacing			
312.6. Nutrition			
312.7. HIV/AIDS (PMTCT)			
312.8. Breastfeeding.			
**313. Advice on danger signs**	Yes=1	No=0	Skip
313.1. Severe headache			
313.2. Abdominal pain			
313.3. Vaginal bleeding			
313.4. A vaginal gush or fluid/ rupture mm			
313.5. Blurred vision.			

Provider-patient interaction observation checklist

S/N	**Provider-patient interaction**	Yes=1	No=0	Skip
314.	While discussing with the women, did the HCP use clear language that the patient understands simply…if the possible local language?			
315.	Did HCP take informed consent before any examination?			
316.	Did HCP take a comprehensive history for pregnant mothers?			
317.	Did the HCP provide a routine physical examination for every woman based on FMOH FANC guidelines?			
318.	Did HCP provide essential counseling for every woman based on FMOH FANC guidelines?			
319.	Did HCP prescribe routine laboratory requests for every woman?			

320.	Did HCP prescribe routine prophylaxis for every woman?			
321.	Did the HCP provide pregnancy danger signs counseling to the women?			
322.	Did the HCP let the patient ask any questions the patient may have?			
323.	Did the HCP respond to the patient's questions?			
324.	Did the HCP stress very well about the problem of defaulting & explain the method they can be easily traced?			
325.	Did the HCP remind the women of the schedule of the next visit?			
326.	Did the HCP record on the unit register immediately after providing the service of every client?			

**Part 4: Mother satisfaction Likert scale items**		V. Satisfied	Satisfied	Neutral	Dissatisfied	V. dissatisfied
401.	How much confidence do you have in being able to get good FANC for you when you need it?	5	4	3	2	1
402.	How satisfied are you with your information on where to get the FANC unit?	5	4	3	2	1
403.	How satisfied are you with the availability of side laboratory?	5	4	3	2	1
404.	How satisfied are you with the availability of essential drugs?	5	4	3	2	1
405.	How satisfied are you with the availability of Equipment?	5	4	3	2	1
406.	How satisfied are you with the appearance of health professionals?	5	4	3	2	1

407.	How satisfied are you with pregnancy danger signs advice?	5	4	3	2	1
408.	How satisfied are you with birth preparedness and complication readiness counseling?	5	4	3	2	1
409.	How satisfied are you with the free service of the entire program?	5	4	3	2	1
410.	How satisfied are you with the overall time spent to get the service?	5	4	3	2	1

Qualitative data collection tools

Part 1: Resource availability.

1.1. Are there program resources in place to deliver FANC services in this Hospital?

1. Yes 2. No

If yes, what do you say about the availability and adequacy of resources for antenatal care? (Probe: trained manpower, drugs, rooms...& countercheck with observation findings)

If no why? ____

1.2. If there is a turnover of HCPs? 1.Yes 2. No

If yes why?

1.3. Were there stock-outs of laboratory reagents (all types) during the last 3 months?

1. Yes 2. No

If yes, for how many days? ____________. Why ____________________

1.4. Had the FANC service been interrupted due to the unavailability of supplies and medicines?

1.Yes 2. No

If yes please specify____

Measurement tools (English version Questionnaires)

Part 2: Protocols and Guidelines availability

2.1. As far as you know, did national or WHO guidelines consistently used in FANC clinic? 1.Yes 2. No

If ye How?

If no, what factors do think are affecting the adherence to national or WHO protocol?

Part 3: Counseling services for pregnant women

3.1. Did HCPs provide counseling for pregnant women regarding health promotion?

If yes how do you offer education? (Individual or group approach), if not why?

Part 4: Management issue for compliance.

4.1. Did your hospital supervise by regional or federal health bureau in 2012 EFY? in the past 6 months? 1. Yes 2. No
If yes, could you please describe how frequently conducted?
If no, why?

4.2. Was there a review meeting organized by the hospital on family health service with a special focus on FANC in 2012 EFY? (Ask for the minutes)

4.3. Is there a continuous quality improvement system in the hospital? Yes/No
If yes, please describe how it is conducted?
If No, why?

4.4. Is there a quality performance review meeting in the hospital? Yes/No

4.5. If yes, how frequently conducted?

4.6. If no, why?

4.7. How is the community involved in the program particularly in quality improvement?

4.8. What are the barriers for the implementation of the FANC program in this hospital?

4.9. Other (specify) _____________
