# Supplementary material for: Evaluation of focused antenatal care services quality at University of Gondar Comprehensive Specialized Hospital, Central Gondar zone, Northwest Ethiopia
Source: PLoS One. 2024 Oct 31;19(10):e0310038. doi: 10.1371/journal.pone.0310038 (PMC11527168; doi:10.1371/journal.pone.0310038)
Supplement: S5 File — (PDF) [file pone.0310038.s007.pdf]

ጎንደር ዩኒቨርሲቲ
የሕብረተሰብ ጤና አጠባበቅ ተቋም
ጎንደር፡ ኢትዮጵያ

University of Gondar
Institute of Public Health
Gondar, Ethiopia

College of Medicine & Health Sciences

Ref No / IPH /837/6/2012
Date /13/2/2020

# To Whom It May Concern

Subject: - Ethical Clearance

- **Tibebu Tadesse Fenta** MPH in Monitoring and Evaluation.post graduate student at Institute of Public Health, University of Gondar and to conduct a research entitled **"Process evaluation of quality of focused antenatal care at the University of Gondar comprehensive specialized hospital, central Gondar zone, Northwest Ethiopia**," The Institute of Public Health Research Ethical Review Committee evaluated the proposal and approved as ethically sound research. The very kind co-operation of your organization is of great importance for the success of the research proposal.

With Best Regards!

ዶ/ር ካሳሁን ዓለሙ
Kassahun Alemu,PhD
የሕብረተሰብ ጤና አጠባበቅ ተቋም ዳይሬክተር
Director, Institute of Public Health

ጎንደር ዩኒቨርሲቲ
University of Gondar
የሕብረተሰብ ጤና አጠባበቅ ተቋም
Institute of Public Health
College of Medicine & Health Sciences

የፖ.ሣ.ቁ
P.O. Box 196
ጎንደር ኢትዮጵያ
Gondar, Ethiopia

ቴሌግራም ጤ.ኮ
Cable A.A.U. PH.
251-058 114 1233
251-058 114 1235
URL.Address:- www.ugondar.edu.et

ስልክ
Telephone PBX 058 111 0174
V/P/ for Academic & Research 058 114 1236
V/President for Admin. 058 114 1238
Human Resource 058 111 0157

መልስ ሲጽፉልን የእኛን ቁጥር ይጥቀሱ።
In Replying, please Quote our Ref. No

B.D/ባ.ደ
